# Resistance mechanisms of tigecycline in *Acinetobacter baumannii*

**DOI:** 10.3389/fcimb.2023.1141490

**Published:** 2023-05-09

**Authors:** Chunli Sun, Yunsong Yu, Xiaoting Hua

**Affiliations:** ^1^ Zhejiang University-University of Edinburgh (ZJU-UoE) Institute, Zhejiang University, Haining, Zhejiang, China; ^2^ Department of Infectious Diseases, Sir Run Run Shaw Hospital, Zhejiang University School of Medicine, Hangzhou, China; ^3^ Key Laboratory of Microbial Technology and Bioinformatics of Zhejiang Province, Hangzhou, China; ^4^ Regional Medical Center for National Institute of Respiratory Diseases, Sir Run Run Shaw Hospital, School of Medicine, Zhejiang University, Hangzhou, China

**Keywords:** *Acinetobacter baumannii*, resistance mechanism, resistance, tigecycline, antibiotic

## Abstract

*Acinetobacter baumannii* is widely distributed in nature and in hospital settings and is a common pathogen causing various infectious diseases. Currently, the drug resistance rate of *A. baumannii* has been persistently high, showing a worryingly high resistance rate to various antibiotics commonly used in clinical practice, which greatly limits antibiotic treatment options. Tigecycline and polymyxins show rapid and effective bactericidal activity against CRAB, and they are both widely considered to be the last clinical line of defense against multidrug resistant *A. baumannii*. This review focuses with interest on the mechanisms of tigecycline resistance in *A. baumannii*. With the explosive increase in the incidence of tigecycline-resistant *A. baumannii*, controlling and treating such resistance events has been considered a global challenge. Accordingly, there is a need to systematically investigate the mechanisms of tigecycline resistance in *A. baumannii*. Currently, the resistance mechanism of *A. baumannii* to tigecycline is complex and not completely clear. This article reviews the proposed resistance mechanisms of *A. baumannii* to tigecycline, with a view to providing references for the rational clinical application of tigecycline and the development of new candidate antibiotics.

## 1 Background

*Acinetobacter baumannii* is a nonfermenting gram-negative bacterium that is one of the most important members of the ESKAPE (*Enterococcus faecium, Staphylococcus aureus, Klebsiella pneumoniae, Acinetobacter baumannii, Pseudomonas aeruginosa*, and *Enterobacter* spp.) group of microorganisms. It is an internationally notorious hospital pathogen, posing a serious threat to public health (Antimicrobial Resistance, 2022). It has been reported that *A. baumannii* is closely related to the occurrence and development of ventilator-associated pneumonia, wound and urinary tract infections, bloodstream infections, endocarditis and meningitis, with very high morbidity and mortality, especially in intensive care unit (ICU) patients (Moubareck and Halat, 2020; Ibrahim et al., 2021; Tokur et al., 2022).

Due to multiple factors, such as irrational use of antibiotics, cross-infection among inpatients and the transmission of resistance genetic elements, an increasing number of strains of *A. baumannii* have evolved into multidrug resistant (MDR), extensive drug resistant (XDR) and even pandrug resistant (PDR) strains (Ibrahim, 2019), which limits the clinical treatment options of antibiotics. At present, carbapenems are commonly used for the treatment of *A. baumannii*; however, in recent years, carbapenem-resistant *A. baumannii* (CRAB) has been widely reported (Zhu et al., 2022).

**Figure 1:**
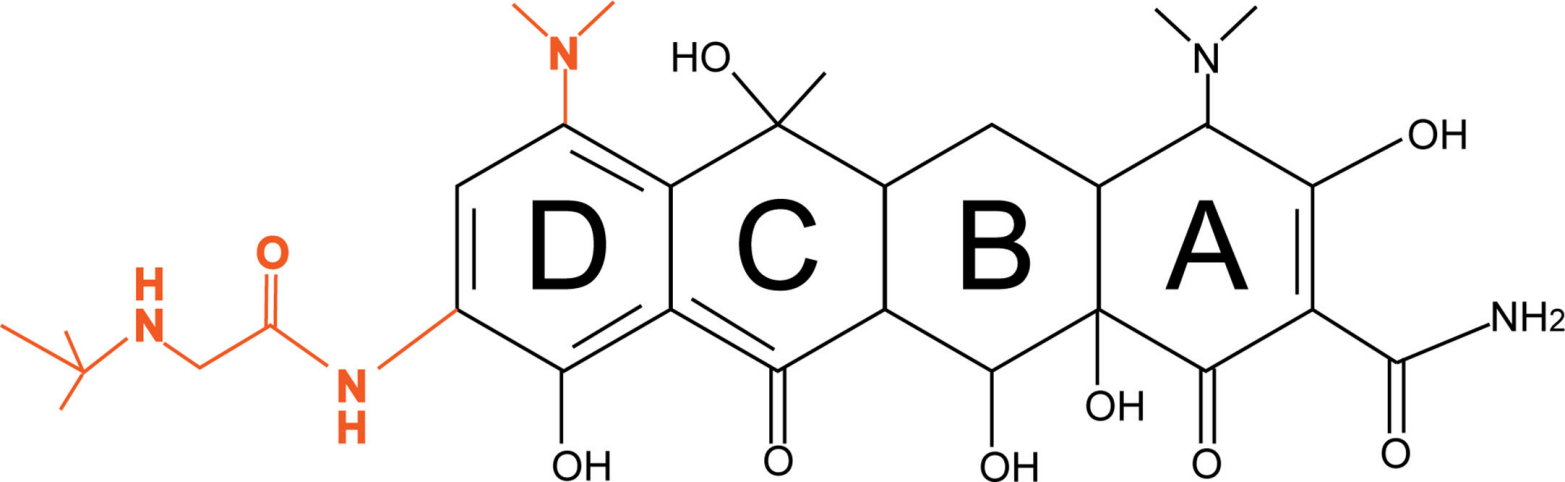
Diagrams of the chemical structures of tetracycline and tigecycline. The chemical structure of tetracycline is shown in black; the only difference between tigecycline and tetracycline is the attachment of 7-dimethylamino and 9-t-butylglycylamido moieties to the D ring, shown in orange.

Tigecycline is a third-generation tetracycline derivative, and the main difference between tetracycline and tigecycline is that ring D of tigecycline is linked to 7-dimethylamido and 9-t-butylglycylamido moieties (**Figure 1**). Due to the stacking interaction of 9-t-butylglycylamido moieties with C1054 of 16S rRNA, tigecycline has an increased affinity for ribosomes, thereby overcoming TetM-mediated tetracycline resistance and exhibiting superior antibacterial activity (Jenner et al., 2013). In addition, the unique chemical structure of tigecycline increases the lipid solubility of the drug and also prevents it from being pumped out of the cell by most membrane-bound efflux proteins by creating a steric hindrance. More importantly, tigecycline also overcomes other antibiotic resistance mechanisms such as drug target modification, enzymatic degradation, and DNA gyrase mutations, making it a promising antibacterial drug for a wide range of applications (Šeputienė et al., 2010).

Gamal et al. collected non-reproducible *A. baumannii* from Vietnam and Germany and performed susceptibility testing of 18 drugs, showing ultra-high resistance rates to fosfomycin (96%), chloramphenicol (95%) and cefotaxime (81%); moderate resistance rates to imipenem (24%) and meropenem (24%), which are carbapenem antibiotics; and low resistance rates to tigecycline (12%) and colistin (3%) (Wareth et al., 2020). Meanwhile, Harald et al. reported data from 2014-2016 for tigecycline and eight commonly used antimicrobial drugs in Africa, Asia, Europe, North America and South America. The results showed that carbapenem resistance rates in all regions were higher in *A. baumannii* isolates, at 65.8%. Since FDA and CLSI breakpoints do not apply to tigecycline, this article does not show data on tigecycline resistance rates, but MIC_90_ values of 1-2 mg/L in all regions could indicate the efficient antibacterial ability of tigecycline (Seifert et al., 2018). The first reported appearance of tigecycline-resistant *A. baumannii* was in 2007. A recent prevalence survey of *A. baumannii* showed that tigecycline resistance was less than 5.5% in Korea, India and China (Chen et al., 2023); nevertheless, clinical cases of *A. baumannii* infection resistant to tigecycline are increasing (Sun et al., 2013), and such a dilemma is worrisome and needs to be taken seriously (Navon-Venezia et al., 2007).

The generation and development of tigecycline resistance mechanisms have attracted widespread attention. Analysis of tigecycline resistance and the resistance mechanism of *A. baumannii* can provide theoretical guidance for the clinical formulation of treatment strategies and control of *A. baumannii* outbreaks.

## 2 Resistance mechanisms

**Figure 2:**
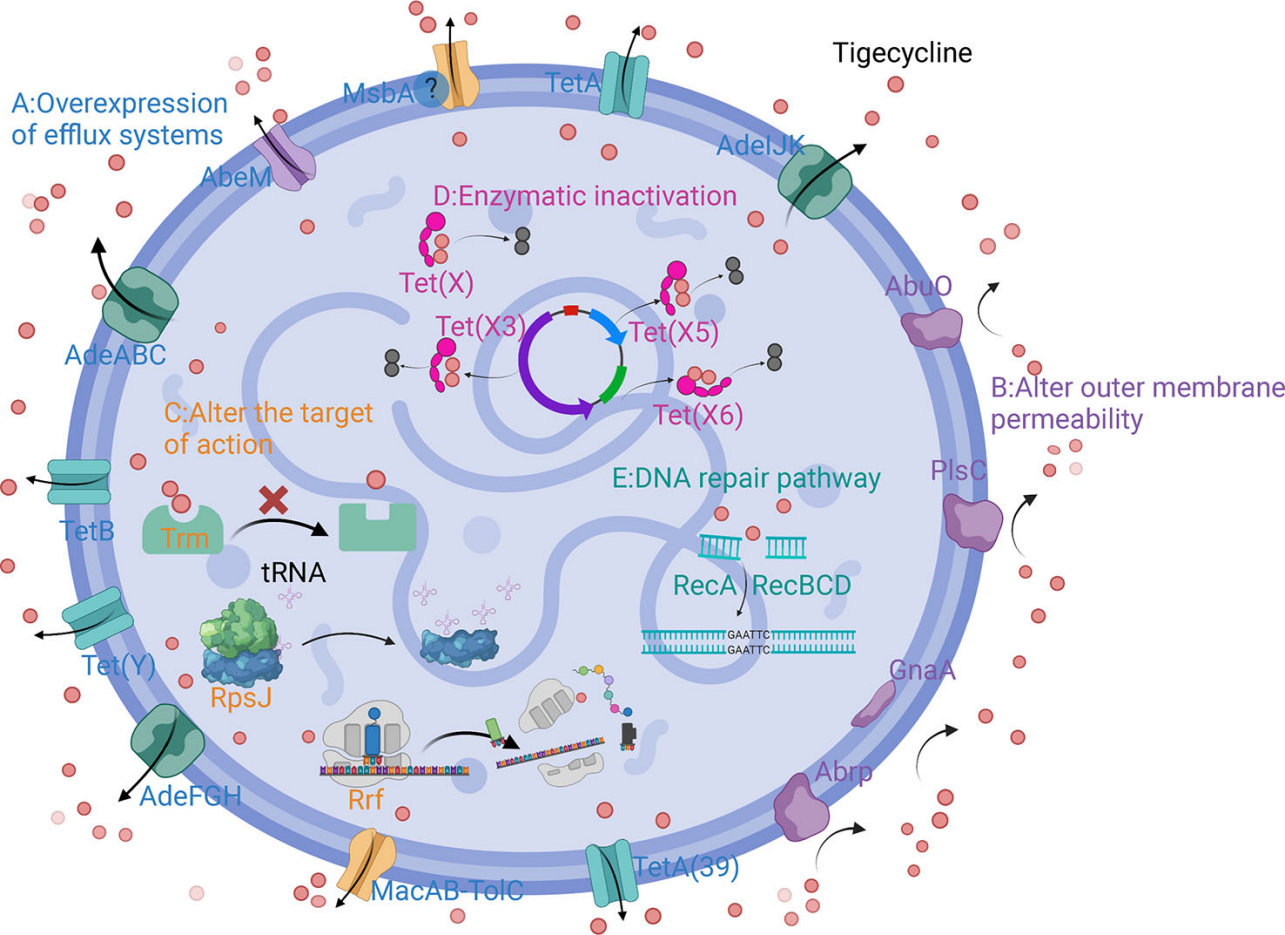
Schematic diagram of the mechanism of tigecycline resistance in *A*. *baumannii*. **(A)** Overexpression of efflux systems; **(B)** Altered outer membrane permeability; **(C)** Altered target of action; **(D)** Enzymatic inactivation; **(E)** DNA repair pathway.

The main mechanisms of tigecycline resistance in *A. baumannii* can be divided into five categories: overexpression of efflux pumps, altered outer membrane permeability, altered tigecycline targets of action, production of tigecycline-inactivating enzymes, and repair pathways mediating tigecycline resistance after DNA damage (**Table 1**) (**Figure 2**).

**Table 1:** Genes associated with tigecycline resistance in *A. baumannii*.

	Gene	Description	Reference
Efflux systems and regulatory factors			
**RND efflux pumps**	*adeABC*	The first RND efflux pump to be studied consisting of *adeA*, *aedB*, and *adeC*	(Magnet et al., 2001)
	*adeFGH*	RND-type efflux pump consisting of *adeF*, *adeG*, and *adeH*	(Coyne et al., 2010b)
	*adeIJK*	RND-type efflux pump consisting of *adeI*, *aedJ*, and *adeK*	(Damier-Piolle et al., 2008)
**MATE family**	*abeM*	MATE pump, an H (+)-coupled multidrug efflux pump	(Su et al., 2005)
**ABC transporters**	*msbA*	ABC transporter, exports lipopolysaccharides from the cytoplasmic membrane to the outer membrane	(Shilling et al., 2006)
	*macAB-TolC*	Prototypical member of Type VII ABC transporter superfamily	(Batista Dos Santos et al., 2022)
**MFS efflux pumps**	*tetA*	Encode the TetA efflux pump	(Jagdmann et al., 2022)
	*tetB*	Encode the TetB efflux pump	(Khlaif and Hussein, 2022)
	*tetA*(39)	Encode the TetA(39) efflux pump	(Rumbo et al., 2013)
	*tet (Y)*	Encode the Tet(Y) efflux pump	(Wang et al., 2021)
**Regulatory factors**	*tetR*	TetR-family transcriptional regulators	(Sumyk et al., 2021)
	*baeSR*	Two-component regulatory system consisting of a sensor histidine kinase (HK) and a cytoplasmic response regulator (RR)	(Leblanc et al., 2011)
	*adeN*	TetR-like transcription regulator	(Rosenfeld et al., 2012)
	*adeL*	LysR-type transcription regulator	(Coyne et al., 2010b)
	*soxR*	global regulator	(Li et al., 2017)
**Outer membrane permeability**	*plsC*	Encode lysophosphatidic acid acyltransferase	(Lu et al., 2005)
	*abrp*	Encode the peptidase C13 family	(Li et al., 2016)
	*gnaA*	Encode a UDP-GlcNAc 6-dehydrogenase	(Wang et al., 2012)
	*abuO*	A TolC-like outer membrane protein	(Srinivasan et al., 2015)
**Antibiotic targets of action**	*rpsJ*	Encode the ribosomal S10 protein	(Beabout et al., 2015)
	*trm*	Encode S-adenosyl-L-methionine-dependent methyltransferase	(Ghalavand et al., 2022)
	*rrf*	Encode ribosome recycling factor	(Ma et al., 2014)
	*rpoB*	RNA polymerase β-subunit	(Cao et al., 2022)
**Antibiotic-inactivating enzyme**	*tet(X)*	Encode flavin-dependent monooxygenase	(Moore et al., 2005)
	*tet(X3)*	Plasmid-mediated Tet(X)-variant Tet(X3)	(He et al., 2019)
	*tet(X5)*	Plasmid-mediated Tet(X)-variant Tet(X5)	(Wang et al., 2019)
	*tet(X6)*	Plasmid-mediated Tet(X)-variant Tet(X6)	(Zheng et al., 2020)
**DNA repair pathway**	*recA*	Major enzyme involved in homologous genetic recombination and recombinational repair	(Aranda et al., 2011)
	*recBCD*	Recombinational repair pathway, repair of DNA double-strand breaks	(Kuzminov, 1999)

### 2.1 Efflux pumps systems

Overexpression of efflux pumps is a key mechanism responsible for drug resistance in *A. baumannii*. Efflux pump excretes antimicrobial drugs from cells, which leads to the decrease of drug concentration and drug resistance (Meyer et al., 2022). There are five superfamilies associated with drug resistance in *A. baumannii*: the resistance-nodulation-cell division (RND) family, multidrug and toxic compound extrusion (MATE) family, the ATP-binding cassette (ABC) transporters, the major facilitator superfamily (MFS) and the small multidrug resistance (SMR) family, of which the first four have been reported to mediate tigecycline resistance (Rafiei et al., 2022).

#### 2.1.1 RND efflux pumps and regulatory factors

**Figure 3:**
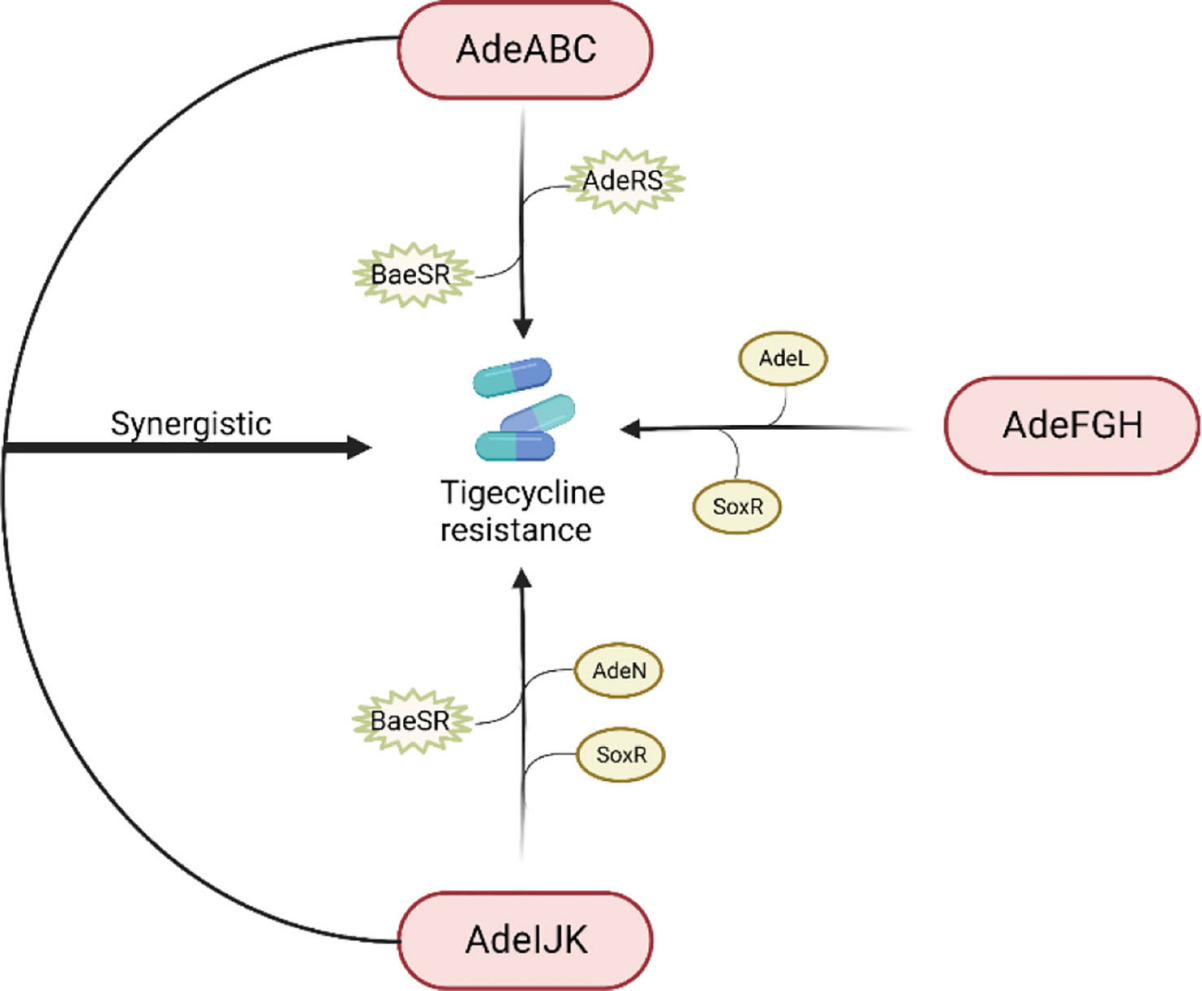
Regulation of tigecycline resistance in *Acinetobacter baumannii* by AdeABC, AdeFGH and AdeIJK efflux pumps. The AdeABC efflux pump is regulated by the two-component system AdeRS and BaeSR; the AdeIJK efflux pump is controlled by the two-component system BaeSR, the global regulator SoxR and the TetR-like transcription regulator AdeN; the expression of AdeFGH is regulated by the LysR-type transcription regulator AdeL and the global regulator SoxR; and AdeABC and AdeIJK are synergistically involved in the production of tigecycline resistance.

**Figure 4:**
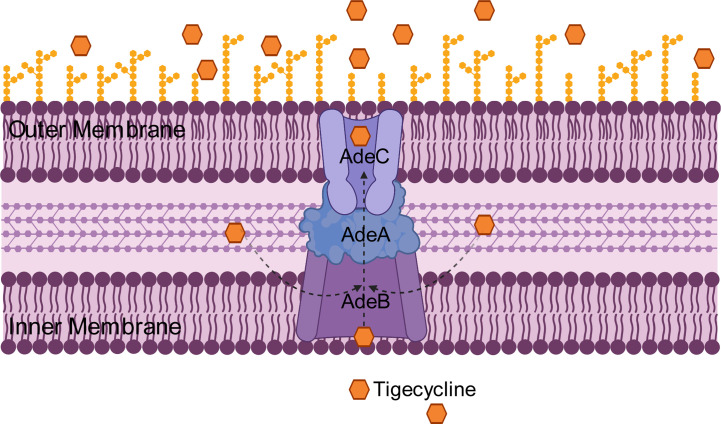
Schematic diagram of the tigecycline resistance mechanism of the AdeABC efflux pump in *A. baumannii*: *adeA* encodes major fusion protein, *adeB* encodes multidrug transporter and *adeC* encodes outer membrane protein, which together form the AdeABC triplet structure. AdeB, assisted by adeA, effluxes tigecycline from the inner membrane or cytoplasm out of the membrane through adeC to exert drug resistance.

The RND efflux pump is the most prevalent efflux pump for MDR *A. baumannii* and contains three major members: AdeABC, AdeFGH and AdeIJK (**Figure 3**). They are all triplets consisting of three structural proteins encoded by structural genes. AdeABC is the first RND system studied in *A. baumannii* and consists of *adeA* (major fusion protein), *adeB* (multidrug transporter) and *adeC* (outer membrane protein) (**Figure 4**) (Coyne et al., 2011). Many previous studies have shown that overexpression of AdeABC contributes to reduced tigecycline susceptibility (Hornsey et al., 2010; Yoon et al., 2013). The expression levels of *adeA*, *adeB* and *adeC* were diverse in different studies. However, most researchers prefer to consider *adeB* as the most important gene in adeABC, as it plays a major resistance role in *A. baumannii* (Yoon et al., 2016). Yin et al. screened over 1000 clinical strains of *A. baumannii* and found that overexpression of the AdeABC efflux pump was the major mechanism for increased tigecycline resistance; furthermore, the expression level of *adeB* showed a linear relationship with the minimum inhibitory concentration (MIC) of tigecycline (Yuhan et al., 2016). Privita et al. demonstrated the essential role of *adeC* in the AdeABC efflux pump by inhibiting *adeC* and achieving complete blockage of the efflux process when designing a potent inhibitor of the AdeABC efflux pump against *A. baumannii* (Verma and Tiwari, 2018). However, in another study, the recombinant *A. baumannii* strain of inactivated *adeC* showed no difference in the efflux function of AdeABC compared to wild-type strains, which seems to indicate that *adeC* is not required to exert resistance (Marchand et al., 2004). Therefore, the role of *adeC* in AdeABC needs to be further explored.

Previous studies have shown that AdeABC expression is regulated by the downstream two-component system AdeRS, which consists of a sensor kinase adeS and a response regulator adeR. Mutations in AdeRS, including A94 V, S8A, H189T, I252S, and T156 M in *AdeS* (Yoon et al., 2013; Lucassen et al., 2021b; Zhang et al., 2022) and P56S, L192R, E219A, and D26 N in *AdeR* (Yoon et al., 2013; Lucassen et al., 2021b), increased the expression level of AdeABC, leading to decreased susceptibility to tigecycline. Jeongwoo et al. (Jo and Ko, 2021) identified the insertion of IS*Aba1* in *adeS* of the tigecycline-resistant subpopulation, and they observed that *adeB* gene expression was upregulated and a soluble truncated adeS protein was produced, which contributed to the overexpression of the AdeABC efflux pump and ultimately caused the tigecycline-resistant outcome, and a similar study also obtained tigecycline-resistant bacteria with IS*Aba1* (Zhang et al., 2022). Another two-component system, BaeSR, has been reported to affect tigecycline susceptibility in *A. baumannii* by positively regulating the *adeA* and *adeB* genes, but the relationship between BaeSR and AdeRS remains unclear (Lin et al., 2014).

Overexpression of AdeFGH was proven to be another mechanism of reduced tigecycline sensitivity in *A. baumannii* and is modulated by AdeL, an encoded LysR-type transcriptional regulator encoded upstream of AdeFGH (Coyne et al., 2010b). However, Deng et al. found no significant difference in the expression levels of the *adeG* gene between tigecycline-resistant and tigecycline-susceptible bacteria (Deng et al., 2014). Therefore, additional tigecycline resistance mechanisms must exist.

Unlike AdeABC and AdeFGH, AdeIJK is present in all *A. baumannii* isolates and leads to intrinsic resistance to tigecycline. The investigators constructed AdeIJK overexpressing bacteria, which resulted in significant inhibition of bacterial growth, and they concluded that AdeIJK pump overexpression inhibits the growth of *A. baumannii* and causes toxic effects (Damier-Piolle et al., 2008). However, Coyne et al. applied RT-PCR and microarray experiments and found that the expression level of AdeIJK in strains with low levels of tigecycline resistance was always lower than that of AdeABC, suggesting that AdeIJK can only be overexpressed in a restricted compartment and then become toxic to the host, which indicates that AdeIJK contributes less to antibiotic resistance. However, the mechanism of AdeIJK toxicity to the host has not been revealed yet and needs to be further elucidated (Coyne et al., 2010a). It has also been shown that AdeN, a transcriptional regulator belonging to the TetR family, is upstream of AdeIJK and can inhibit the expression of AdeIJK (Rosenfeld et al., 2012). In addition, the BaeSR system may also be involved in the regulation of AdeIJK. Transcriptomic data from an LPS-deficient *A. baumannii* strain showed increased expression levels of the *baeS* and *baeR* genes, as well as increased expression levels of the AdeIJK genes (Henry, 2012).

AdeABC, AdeFGH and AdeIJK are implicated in tigecycline resistance in *A. baumannii*. Yoon et al. evaluated the relative contribution of these three efflux pumps in the clinical setting and concluded that AdeABC plays a major role in resistance, while there was no necessary association between the tigecycline MIC and AdeABC expression levels (Yoon et al., 2013). Laurence et al. found that mutants with dual inactivation of the AdeABC and AdeIJK efflux pump genes were much more susceptible to tigecycline than wild-type strains and mutants with inactivation of either single efflux pump gene, suggesting that AdeABC and AdeIJK can be synergistically involved in tigecycline resistance (Damier-Piolle et al., 2008). In 2017, a study from Beijing first proposed that the global regulator SoxR is a negative regulator of efflux pump gene expression. By constructing *soxR* overexpression mutants, they interestingly found that *soxR* overexpression resulted in statistically significant reduced expression levels of *adeJ*, *adeG*, and *adeS* and increased (2-fold) susceptibility to tigecycline, implying that SoxR is involved in tigecycline resistance regulation in *A. baumannii* (Li et al., 2017).

#### 2.1.2 (MATE) family

MATE transporters can drive various toxic and harmful substances out of the membrane through Na^+^ or H^+^ as a coupling ion. It is an important defense mechanism for bacteria to resist external environmental pressure (Zhao et al., 2021). Here, AbeM is an H^+^-coupled multidrug efflux pump that belongs to the MATE transporter family (Su et al., 2005). Recently, Mina et al. reported that *abeM* gene expression was increased 16- to 256-fold in tigecycline-resistant *A. baumannii* compared to controls, and an increase in the *abeM* gene was observed in 70% of CCCP-positive tests, (CCCP is an abbreviation for carbonyl cyanide *m*-chlorophenyl hydrazone, a known pump inhibitor, the purpose of the CCCP-positive test is to screen for potential efflux pumps that are not inhibited by CCCP and play a role in drug resistance), indicating that abeM is involved in tigecycline resistance (Owrang et al., 2018). In contrast, Deng et al. found that tigecycline-resistant *A. baumannii* showed only 1-fold higher *abeM* gene expression levels than sensitive bacteria, and the difference was not significant (Deng et al., 2014). What these inconsistencies can indicate is that the resistance of *A. baumannii* to tigecycline is not unique, and there are additional mechanisms.

#### 2.1.3 ABC transporters

The ATP-binding cassette (ABC) transporter differs from other family transporters in that it uses the energy generated by ATP binding and hydrolysis to perform efflux functions (Okada and Murakami, 2022). MsbA was the first ATP-binding cassette transporter to be crystallized and analyzed (Shilling et al., 2006). Chen et al. transferred the wild-type *msbA* gene into a tigecycline-resistant mutant (*msbA* ^A84V^), but they found no change in MIC, and the mutant strain had a smaller colony size than the parental strain, so they concluded that *msbA* did not contribute to tigecycline resistance but could reduce fitness (Chen et al., 2014). In another study, in contrast, *msbA* had a highly significant p value in the tigecycline-resistant group; they considered *msbA* mutations to be gain-of-function mutations and speculated that a possible resistance mechanism is that these mutations expand pump specificity, facilitating tigecycline efflux (Hammerstrom et al., 2015). Overall, the function of msbA needs to be re-evaluated.

MacAB-TolC is another important efflux pump system involved in tigecycline resistance. Lin et al. reported a statistically significant increase in the expression level of the *macB* gene in tigecycline-resistant strains compared to susceptible strains (Lin et al., 2017). In addition, the BaeSR system regulates macAB-TolC expression (Henry, 2012).

#### 2.1.4 MFS efflux pumps

The MFS efflux pump is a proton-dependent antimicrobial drug efflux system that includes passive transporters and secondary active transport systems (Stephen et al., 2023). Most reports on TetA(39) have been on resistance to tetracycline and doxycycline. The study by Rumbo et al. proposed that tigecycline-resistant *A. baumannii* may be due to a novel efflux pump system, TetA(39) (Rumbo et al., 2013). TetA is an important member of the MFS family, and its expression is controlled by TetR of the TetR-family transcriptional regulators (TFR) (Sumyk et al., 2021). Foong et al. reported that all strains possessing pBAV1K_*TetA*, including adeAB and adeIJ knockout strains, showed increased MICs to tigecycline compared to controls (without the *tetA* gene). Furthermore, when exposed to tigecycline, the expression levels of *tetA* in *A. baumannii AYE* showed remarkable elevation, while *adeB* and *adeJ* genes were expressed at only considerable levels. Their findings demonstrate that TetA is a predominant determinant in tigecycline resistance and synergizes with AdeABC and AdeIJK (Foong et al., 2020). Recently, however, another study reported that all tigecycline-nonsusceptible *A. baumannii* isolates in their collection did not have the *tetA* gene but instead found the *tetB* gene (Khlaif and Hussein, 2022). Like TetA, TetB is also a non-chromosomally encoded MFS efflux pump (Verma et al., 2021). Overall, there is a correlation between TetB and TetA and tigecycline resistance, and more research is urgently needed to reveal the underlying mechanisms.

Tet(Y) originally encodes tetracycline resistance and is a member of the tetracycline-specific efflux pump (Fang et al., 2020). Wang et al. first observed that the plasmid-mediated *tet(Y)* gene is a determinant of tigecycline resistance in *A. baumannii*. They introduced the plasmid containing the *Tet(Y)* gene into the host bacterium, resulting in a 4-fold increase in tigecycline MIC. Moreover, they found that 2016GDAB1 (The *tet(Y)* and *tetA*(39) genes coexist on the p2016GDAB1 plasmid of *A. baumannii*, both of which are followed by a *tetR* gene) showed a 128-fold increase in MIC, suggesting that *tet(Y)* and *tetA*(39) together synergistically confer high levels of tigecycline resistance (Wang et al., 2021).

### 2.2 Outer membrane permeability

To prevent the entry of antimicrobial agents into cells, reducing the permeability of the outer membrane is another common mechanism of resistance.

*PlsC*, encoding 1-acyl-sn-glycero-3-phosphate acyltransferase, catalyzes the acylation of lysophosphatidic acid to form phosphatidic acid, thereby playing a role in maintaining epidermal permeability barrier function (Lu et al., 2005). Li et al. identified a strain of *A. baumannii*, 19606-M24, with high levels of resistance to tigecycline (MIC = 24 mg/L), and whole genome comparison revealed a mutation in *plsC*. Complementation studies revealed that strains introduced with the wild-type *plsC* gene restored susceptibility to tigecycline, indicating that the frameshift mutation of *plsC* gene is associated with tigecycline resistance. In subsequent flow cytometry, the highest membrane potential was observed for 19606-M24, while a decrease in membrane potential was also observed in complementary strains. These results convincingly demonstrate that *plsC* mediates resistance to tigecycline by affecting cell membrane permeability (Li et al., 2015).

Soon thereafter, Li et al. identified a new gene, *abrp*, encoding the peptidase C13 family, with a frameshift mutation. Compared to the *abrp* knockout strain, the wild-type strain showed reduced susceptibility to a variety of antibiotics, including tigecycline. Furthermore, they found that the *abrp* deletion strain increased cell membrane permeability accompanied by impaired bacterial growth. Thus, it can be concluded that *abrp* increases resistance to tigecycline by altering cell membrane permeability (Li et al., 2016).

Another gene, *gnaA*, has also been reported to be associated with tigecycline resistance (Hammerstrom et al., 2015; Lucassen et al., 2021a). *GnaA* encodes an enzyme involved in catalyzing the initial step of UDP-D-MANPNAC3NACA synthesis and was first identified in *Escherichia coli* (Wang et al., 2012). In 2019, Xu et al. identified tigecycline-resistant mutant strains with an insertion of ISAba16 in the *gnaA* gene of unknown function. The membrane potential of *gnaA*-deficient bacteria was significantly higher than that of the wild-type strain, and complementation with the wild-type *gnaA* gene resulted in a reduced membrane potential, indicating that the barrier function of the cell membrane was restored. Moreover, the researchers revealed that the *gnaA* gene affects lipooligosaccharide (LOS) and capsular polysaccharide (CPS) synthesis and that disruption of *gnaA* also affects the virulence, morphology, and susceptibility of *A. baumannii* to other classes of antibiotics. Unfortunately, no changes in tigecycline MIC were seen in *gnaA* complementary experiments, this could still suggest that the *gnaA* gene may be involved in tigecycline resistance by altering cell membrane permeability (Xu et al., 2019). In conclusion, the resistance of *gnaA* to tigecycline needs to be supported by more studies.

Outer membrane protein (OMP) is a specific channel protein present in the lipid bilayer structure of gram-negative bacteria, located on the surface of the cell membrane or embedded in it, which can serve as a channel for antibiotics to enter the cell (Tokuda, 2009). Vijaya et al. uncovered a putative OMP, abuO, associated with antimicrobial and oxidative stress resistance in *A. baumannii*. Compared with the wild-type strain, the *abuO* knockout strain showed increased susceptibility to different types of antibiotics, including tigecycline, with MIC values changing from 2 mg/L to 0.75 mg/L. Meanwhile, the MIC of the complementary *abuO* isolate again returned to the same level as the wild-type strain. This may indicate a role for *abuO* in tigecycline resistance (Srinivasan et al., 2015). In addition, RT−PCR results showed that the *abuO*-deficient strain not only increased the expression levels of efflux pump-related genes but also changed the expression of other membrane proteins, which implies that abuO plays an integral role in *A. baumannii* and that its relationship with tigecycline resistance mechanisms should still be investigated in depth.

### 2.3 Antibiotic targets of action

Changing the target of antibiotic action is also one of the mechanisms by which bacteria develop resistance. Bacteria prevent antibiotic binding by altering the target structure, thereby creating resistance.

The S10 protein, which is closest to the tigecycline binding pocket, consists of 53-60 amino acid residues encoded by the *rpsJ* gene and helps to participate in maintaining the normal structure of the tigecycline binding site. Researchers found that *rpsJ* mutations emerged in a variety of pathogens under tigecycline exposure and conferred resistance to tigecycline. They believe that mutations in *rpsJ* caused a structural change in the S10 loop, which in turn changes the conformation of the 16S RNA, ultimately leading to reduced binding affinity for tigecycline and ribosomes. Another model is that reduced tigecycline susceptibility promotes entry of tRNA and increases binding to ribosomes (Beabout et al., 2015). Additionally, since *rpsJ* mutations have a low fitness cost and occur widely in a variety of pathogens, all tested strains had a mutation at amino acid position 57 of S10, while gram-positive bacteria have a higher variation at amino acid position 60 (**Figure 3**). These results suggest that *rspJ* has promise as an important marker for detecting whether pathogens are resistant to tigecycline. Another result likewise reported that *rspJ* plays an important role in tigecycline resistance (Hammerstrom et al., 2015).

The gene *trm* (tigecycline-related-methyltransferase) encodes S-adenosyl-L-methionine-dependent methyltransferase. Chen et al. obtained the resistant strain 19606-T8 (MIC = 8 mg/L) with a deletion mutation in the *trm* gene and then transformed the wild-type *trm* gene into 19606-T8. Interestingly, it restored the sensitivity to tigecycline, MIC dropping from 8 mg/L to 1 mg/L, implying that *trm* plays an important role in reducing *A. baumannii* susceptibility to tigecycline (Chen et al., 2014). However, how methyltransferase mediates the reduction in sensitivity to tigecycline has not been investigated. Another study also reported diverse amino acid alterations in *trm* of tigecycline-resistant isolates, such as S94A, V121A, C285F, K291R, G310C, H312P, T321I, T323S, and M378K (Ghalavand et al., 2022). The mechanism by which trm mediates resistance to tigecycline is unclear. As a methyltransferase, trm may act as ArmA, RmtA and NpmA to methylate the ribosomal target, hinder the binding of tigecycline to its target, and mediate tigecycline resistance (Fritsche et al., 2008; Chen et al., 2014).

The *rrf* gene encodes the production of ribosome recycling factor (RRF). RRF is an essential component of protein synthesis and is involved in the release of polypeptides from the ribosome for a new round of translation (Ma et al., 2014). In an earlier study, Hammerstrom et al. identified mutations in the *rrf* gene in tigecycline-resistant *A. baumannii*. They proposed that mutations in *rrf* (M1 V, N3N) might reduce translation of mRNA and in return affect the binding of tigecycline to the ribosome, but they did not proceed to verify this hypothesis (Hammerstrom et al., 2015).

Subsequently, Hua et al. revealed the mechanism of *rrf* involvement in tigecycline resistance. They constructed recombinant mutants XH1457 (MDR-ZJ06 rrf^H33P^) and XH1458 (MDR-ZJ06 rpoB^G136D^), which showed only a slight increase in tigecycline tolerance compared to the parental strain, but Raman spectrometry provided a strong complement to the tigecycline resistance conferred by these two mutant genes. Furthermore, it was confirmed that mutations in *rrf* decreased the expression of RRF proteins, affecting the ribosome recycling process and ultimately exhibiting a tigecycline-resistant phenotype. In addition, the transcriptomic data also revealed that *rpoB* ^G136D^ could regulate the expression level of *trm* (Hua et al., 2021).

### 2.4 Antibiotic-inactivating enzyme

Resistance to antibiotics by degrading and modifying antibiotics to make them inactive is an important resistance mechanism for bacteria.

Several studies have recently reported the widespread dissemination of Tet(X) and its variants through plasmid mediation in *A. baumannii*, giving rise to resistance to a variety of tetracyclines, which has drawn the attention of researchers. Tet(X) encodes flavin-dependent monooxygenase that modifies first- and second-generation tetracycline. Tigecycline is one of the substrates of Tet (X), which requires activation in the presence of FAD, NADPH, O_2_, and Mg2^+^, Tet (X) modifies tigecycline to 11a-hydroxytigecycline, which alters the physical properties of tigecycline by weakening its binding to magnesium and decreasing its affinity to ribosomes, this inactivates tigecycline, thus allowing bacteria to develop resistance (Moore et al., 2005).

To date, eight genes associated with high levels of resistance to tigecycline have been reported in the Tet(X) family, namely Tet(X), Tet(X1), Tet(X2), Tet(X3), Tet(X4), Tet(X5), Tet(X6), Tet(X7), where Tet(X1) is a truncated variant with no enzymatic activity. These Tet(X) and its variants have been detected in various pathogens such as *Klebsiella pneumoniae, Pseudomonas aeruginosa and Escherichia coli*. Among them, Tet(X), Tet(X3), Tet(X5), and Tet(X6) have been reported to confer tigecycline resistance in *A. baumannii (* Cui et al., 2021). In addition, the Tet(X) was originally identified in the *obligate anaerobe Bacteroides fragilis (* Yang et al., 2004). As reported in the literature, compared to Tet(X) (M37699), Tet(X3) (MK134375), Tet(X5) (CP040912), Tet(X6) (CP044517) showed 86%, 90%, 84% amino acid identities, respectively (Cui et al., 2021). He et al. demonstrated that Tet(X3) catalyzes the oxygenation of tigecycline at C11a to form 11a-hydroxyltigecycline, resulting in tigecycline inactivation. Whereas the MIC of tigecycline was substantially increased (64-fold) in strains possessing the Tet(X3)-carrying plasmid, mouse infection models likewise revealed that Tet(X3) impaired the therapeutic efficacy of tigecycline *in vivo* and ultimately led to drug resistance (He et al., 2019). Wang et al. identified another Tet(X) variant, Tet(X5), which is structurally and functionally similar to other variants and hydroxylates tigecycline. Tet(X5) shows increased resistance to a wide range of tetracyclines (Wang et al., 2019). Not coincidentally, scientists have also found that Tet(X5) and Tet(X6) in *A. baumannii* are resistant to tigecycline through hydroxylation and that ISCR2-mediated transposition and recombination exacerbate the spread of Tet(X5) (Chen et al., 2021). Notably, IS*CR2* is an insertion sequence type element capable of transposing neighboring DNA sequences, including antibiotic resistance genes, through a process of rolling-circle transposition (Chen et al., 2021). IS*CR2* shares 65% amino acid identity with IS*CR1*, which is a mobile element involved in the spread of resistance genes (Xu et al., 2017).

Overall, the clonal transmission of Tet(X) and its variants should not be underestimated and deserves attention.

### 2.5 DNA repair pathway

It has been demonstrated that DNA damage under antibiotic induction can mediate the killing of *A. baumannii* (Sampson et al., 2012), and recA and recBCD are important pathways for DNA repair. RecA is the major enzyme involved in gene homologous recombination and recombination repair (Aranda et al., 2011), and recBCD is involved in DNA double-stranded repair (Kuzminov, 1999). Taofeek et al. found increased susceptibility of recA- and recBCD-inactivated *A. baumannii* to various antibiotics, including tigecycline, suggesting that the recA- and recBCD-mediated repair pathway may protect *A. baumannii* from antibiotic killing, leading to drug resistance (Ajiboye et al., 2018).

### 2.6 Other potential mechanisms

In addition to the abovementioned mechanisms related to tigecycline resistance in *A. baumannii*, there are some potential resistance genes that need to be further investigated. Nogbou et al. found that *A. baumannii* with mutations in both the *parC* and *gyrA* genes had higher levels of resistance to tigecycline than strains without mutations (Nogbou et al., 2021). Hammerstrom et al. used a bioinformatics approach to infer that *rnpA*, *rpoD*, *rppH*, *wzc*, and *pcaF* are candidate tigecycline resistance genes (Hammerstrom et al., 2015). Whether these genes can indeed mediate tigecycline resistance and by what mechanism they cause resistance needs to be investigated more thoroughly in the future.

## 3 Conclusions

Tigecycline-resistant *A. baumannii* is a global public health terrorist. To adapt to the pressures of the external environment, bacteria have evolved multiple resistance strategies. Many studies have shown that overexpression of the RND efflux pump is the main cause of tigecycline resistance in *A. baumannii* and that the expression level of the efflux pump-related gene *adeB* is positively correlated with the MIC value of tigecycline (Yuhan et al., 2016), but some researchers have reported that there is no necessary link between the two (Yoon et al., 2013). The expression of the efflux pump is also regulated by various regulatory factors, and the efflux pumps can interact with each other. Therefore, the mechanism of the active efflux pump is diverse and complex, and a comprehensive study of the mechanism of action of the efflux pump is needed.

The lipid bilayer is an important barrier for *A. baumannnii*, and loss of channel proteins or genetic mutations that reduce outer membrane permeability can lead to difficulties in tigecycline entry into the cell and an increase in the minimum inhibitory concentration of bacteria. Therefore, focusing on weakening lipopolysaccharides bilayer synthesis to make bacteria sensitive to antibiotics is the conventional direction of thinking. In addition, a deeper understanding of the function and properties of channel proteins, as well as the identification of regulatory factors that may affect outer membrane permeability and channel protein closure, will facilitate the targeted development of novel drugs and improve current clinical drug therapies.

As described above, *rpsJ* and *rrf* can be mutated to reduce the ability to bind ribosomal proteins (Beabout et al., 2015; Hammerstrom et al., 2015; Hua et al., 2021), while *trm* may alter the site of action of the drug through enzymatic modifications, all of which ultimately lead to resistance to tigecycline (Ghalavand et al., 2022). Importantly, the high-frequency site of tigecycline resistance due to *rpsJ* mutations is amino acid position 57, which occurs not only in *A. baumannii* but also in a wide range of pathogens (Beabout et al., 2015). This provides a unique way to identify tigecycline resistance by screening for *rpsJ* mutations in the future.

Tet(X) can inactivate tigecycline and develop resistance. Worryingly, novel Tet (X) variants continue to be discovered and they can spread rapidly through horizontal gene transfer in a wide range of pathogens and appear to be on the verge of a global epidemic (Fang et al., 2020). Recently, Tet(X) and its variants have been found to confer high levels of tigecycline resistance and cause clonal transmission in different species of bacteria through plasmid-mediated and widespread association with other resistance genes and transposons such as blaOXA-72, blaOXA-58, blaNDM-1, and IS*CR2*, posing a great safety risk to the health care system (Zheng et al., 2020; Chen et al., 2021; Hsieh et al., 2021). This calls for effective measures to intervene in the occurrence of such epidemic events. For example, we need to investigate in depth the origin, structure and evolutionary mechanisms of *Tet(X)* variants, as well as design inhibitors and disruptors against this enzyme to overcome the high levels of tigecycline resistance mediated by *Tet(X)* and variants.

RecA and recBCD are important factors involved in the repair of DNA after damage. RecA was shown to protect *A. baumannii* from UV light, heat shock, desiccation and several classes of antibiotics including tigecycline in the study by Aranda et al. In addition, *in vitro* and *in vivo* virulence assays showed that the virulence of the *A. baumannii* recA mutant was significantly reduced. Another study similarly confirmed that recA and recBCD deletions contribute to tigecycline-mediated lethality against *A. baumannii*. Accordingly, future attention could be focused on the inhibition of recA, recBCD for the purpose of treating *A*. baumannii infections.

In summary, *A. baumannii* can produce resistance to tigecycline through RND efflux pumps (AdeABC, AdeFGH, AdeIJK) (Magnet et al., 2001; Damier-Piolle et al., 2008; Coyne et al., 2010b), MATE family (AbeM) (Su et al., 2005), ABC transporters (MsbA, MacAB-TolC) (Shilling et al., 2006; Batista Dos Santos et al., 2022), and MFS efflux pumps (TetA, TetB, TetA(39), Tet (Y)) (Rumbo et al., 2013; Wang et al., 2021; Jagdmann et al., 2022; Khlaif and Hussein, 2022). In addition, *A. baumannii* also reduces membrane permeability (plsc, abrp, gnaA, abuO) (Lu et al., 2005; Wang et al., 2012; Srinivasan et al., 2015; Li et al., 2016), alters antibiotic targets (rpsJ, trm, rrf, rpoB) (Ma et al., 2014; Beabout et al., 2015; Hua et al., 2021; Ghalavand et al., 2022), produces tigecycline inactivating enzymes (Tet(X) and its variants Tet (X3), Tet(X5), Tet(X6)) (Moore et al., 2005; He et al., 2019; Chen et al., 2021) and DNA repair pathways (recA and recBCD) (Kuzminov, 1999; Aranda et al., 2011) mediating tigecycline resistance. In addition, these resistance mechanisms can be regulated by various regulatory factors.

As described in the review, the resistance mechanisms of *A. baum*annii to tigecycline are diverse and complex, and the emergence of resistance is also very complex. Different A. baumannii cause tigecycline resistance by different mechanisms, and several resistance mechanisms can even coexist in the same bacterium. These resistance mechanisms can also be regulated by various regulatory factors. By screening for resistance-associated genetic mutations to identify tigecycline resistance and applying targeted inhibitors against *A. baumannii* infection according to these described resistance mechanisms, we hope to provide clinicians with scientific treatment schemes.

## Author contributions

CS, XH, and YY conceived the review, CS wrote the manuscript. XH critically revised the manuscript. All authors contributed to the article and approved the submitted version.
